# Operation of a P300-based brain-computer interface in patients with Duchenne muscular dystrophy

**DOI:** 10.1038/s41598-018-20125-6

**Published:** 2018-01-29

**Authors:** Kota Utsumi, Kouji Takano, Yoji Okahara, Tetsuo Komori, Osamu Onodera, Kenji Kansaku

**Affiliations:** 1 Systems Neuroscience Section, Department of Rehabilitation for Brain Functions, Research Institute of National Rehabilitation Center for Persons with Disabilities, Tokorozawa, Saitama, 359-8555 Japan; 2 Department of Neurology, Brain Research Institute, Niigata University Graduate School of Medicine, Niigata, Niigata, 951-8585 Japan; 3 Department of Neurological Surgery, Chiba University Graduate School of Medicine, Chiba, Chiba, 260-8670 Japan; 4 Department of Neurology, National Hakone Hospital, Odawara, Kanagawa 250-0032 Japan; 5 Brain Science Inspired Life Support Research Center, The University of Electro-Communications, Chofu, Tokyo, 182-8585 Japan

## Abstract

A brain-computer interface (BCI) or brain-machine interface is a technology that enables the control of a computer and other external devices using signals from the brain. This technology has been tested in paralysed patients, such as those with cervical spinal cord injuries or amyotrophic lateral sclerosis, but it has not been tested systematically in Duchenne muscular dystrophy (DMD), which is a severe type of muscular dystrophy due to the loss of dystrophin and is often accompanied by progressive muscle weakness and wasting. Here, we investigated the efficacy of a P300-based BCI for patients with DMD. Eight bedridden patients with DMD and eight age- and gender-matched able-bodied controls were instructed to input hiragana characters. We used a region-based, two-step P300-based BCI with green/blue flicker stimuli. EEG data were recorded, and a linear discriminant analysis distinguished the target from other non-targets. The mean online accuracy of inputted characters (accuracy for the two-step procedure) was 71.6% for patients with DMD and 80.6% for controls, with no significant difference between the patients and controls. The P300-based BCI was operated successfully by individuals with DMD in an advanced stage and these findings suggest that this technology may be beneficial for patients with this disease.

## Introduction

A brain-computer interface (BCI) or brain-machine interface (BMI) is a technology that enables the control of computers and other external devices without requiring any muscle movements, and that instead uses electrical signals from the brain, obtained by non-invasive or invasive means^1,2^. Invasive BCI acquires electrical signals directly from neurons after a surgical operation^3,4^, whereas non-invasive BCI acquires an electroencephalogram (EEG) from external scalp electrodes. Slow cortical potential (SCP), sensorimotor rhythms (SMR), steady-state visual evoked potentials (SSVEP), and P300^5–8^ have been used for non-invasive BCI; indeed, P300 is one of the main BCI approaches to providing effective communication^9^.

P300 is an event-related potential related to “attention,” and can be induced by the oddball paradigm, which is reproduced by randomly presenting two or more types of physical stimuli at different frequencies. When subjects attend to low- frequency stimulation and make a predetermined reaction, P300 occurs after the onset of the stimulus, regardless of the type of physical stimulus, such as visual, auditory, or tactile. P300 reflects higher-order cognitive responses to unexpected stimuli and cognitively important stimuli^10,11^.

Farewell and Donchin applied such elicited P300 responses to a BCI speller. They used a visual oddball task by allocating alphabetic characters to a matrix arranged in 6 rows × 6 columns (RC speller), and the users were asked to gaze at one of the icons that was intensified. EEG responses were analysed to determine the target icon^12^. Such a P300 BCI speller has been applied to patients with motor dysfunction, such as spinal cord injury^13^ and brain stem infarction^14^. In particular, amyotrophic lateral sclerosis (ALS), a neurodegenerative disease that causes progressive paralysis, has been one of the main diseases for patient studies of the BCI speller^15–19^. Recently, our research group developed a region-based two-step P300 speller, which has a larger flashing area than the visual array of the conventional P300 speller. We then showed that the two-step procedure for the visual P300 BCI system provided significantly increased accuracy for ALS patients, compared with a conventional RC speller^20^.

McDonald showed that muscle weakness and muscle fatigue were important factors that adversely affected the quality of life in neuromuscular disease patients^21^. ALS is a well-known neuromuscular disease, but there are other neuromuscular diseases. Duchenne muscular dystrophy (DMD) is a neuromuscular diseases characterised by skeletal muscle atrophy of the whole body affecting proximal muscles and causing gait disturbance, due to the loss of the dystrophin-encoding gene, which encodes one of the most important proteins in myocyte membranes^22^. The DMD patients ultimately progress to a bedridden state requiring full assistance. In the past, their average life span was considered to be about 20 years, primarily because of respiratory failure, but due to advances in respiratory management, that life span has now increased to around 30^23,24^.

Patients with DMD show muscle weakness and muscle fatigue. After undergoing a tracheostomy, it becomes difficult for them to communicate verbally. These patients do not usually show deterioration in cognitive function and a worsening of consciousness level is associated with progression of the medical condition. Patients with DMD often use alternative augmentative communication (AAC) devices, but muscle weakness and muscle fatigue make it hard to use them continuously. Thus, it is desirable that patients with DMD also have the option of using communication devices that do not depend on muscle activity. Thus, patients with DMD may be considered as candidate BCI users, but BCI technology has not yet been tested systematically with these patients.

In this study, we investigated the efficacy of a P300-based BCI for patients with DMD. Bedridden patients with DMD and age- and gender-matched able-bodied controls were instructed to input hiragana characters using a region-based, two-step P300-based BCI. Here, we show that the P300-based BCI was operated by individuals with DMD at an advanced stage.

## Results

**Figure 1:**
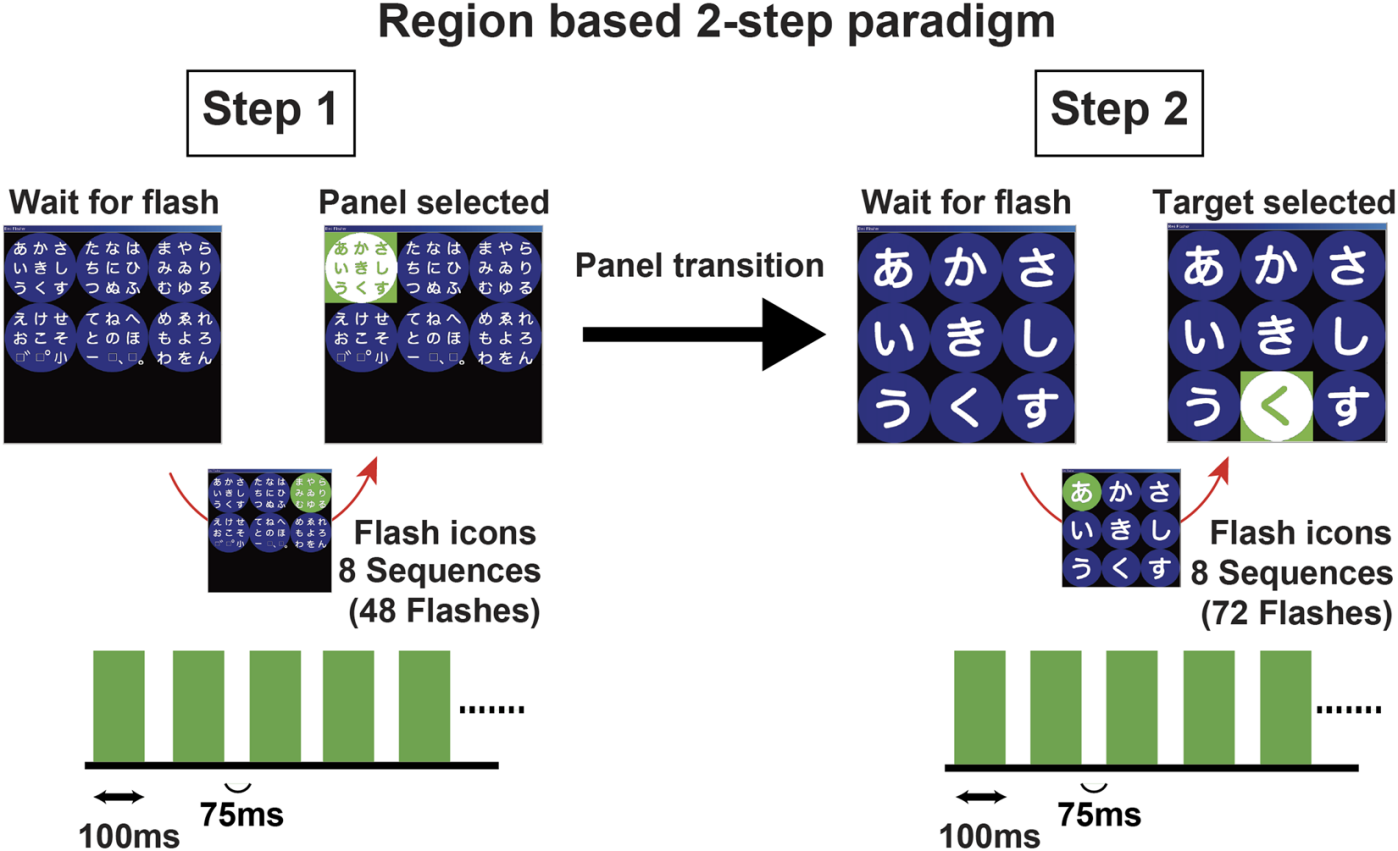
A region-based two-step P300-based hiragana speller. The subject was required to count the number of intensifications of the green/blue circle containing the target character to be input. Each circle flashed eight times, and the interval between two flashes was 175 ms, consisting of 100 ms of intensification (green) and 75 ms of rest (blue). We divided the 6 × 9 matrix into six circled regions including nine characters each. First, each region was intensified individually. When a region that included a target character was selected, the speller matrix moved to the second step. The second step used 3 × 3 regions with one character each, and each region was again intensified.

We used a region-based two-step P300-BCI speller (Fig. 1)^20^ to compare the accuracy of hiragana character input in a DMD patient group and an age- and gender-matched able-bodied participant group. Eight patients with DMD (age: 23–38 years, mean: 30.3), diagnosed by genetic testing and proof of dystrophin deficiency, were recruited as participants (Table 1). These patients had experienced walking disabilities before reaching 5 years old, and their current level of activities of daily living (ADL) was at the total assistance level; their modified Rankin Scale score was 5. Online and offline performances in both groups were evaluated.

**Table 1:** Demographic and clinical characteristics of the patients with DMD.

No	Age	Gender	mRs	Tracheostomy	Communication	Genetic deletion
1	33	male	5	+	lip-reading/visual scanning board	exon 48–50
2	28	male	5	+	lip-reading/visual scanning board	exon 18–44
3	28	male	5	−	speech	exon 51
4	27	male	5	−	speech	exon 48–50
5	23	male	5	−	speech	exon 48–52
6	38	male	5	+	lip-reading/visual scanning board/ AAC device	exon 7–9
7	36	male	5	+	lip-reading/visual scanning board	unspecified
8	29	male	5	+	lip-reading/visual scanning board	unspecified

### Performance in BCI operation

**Figure 2:**
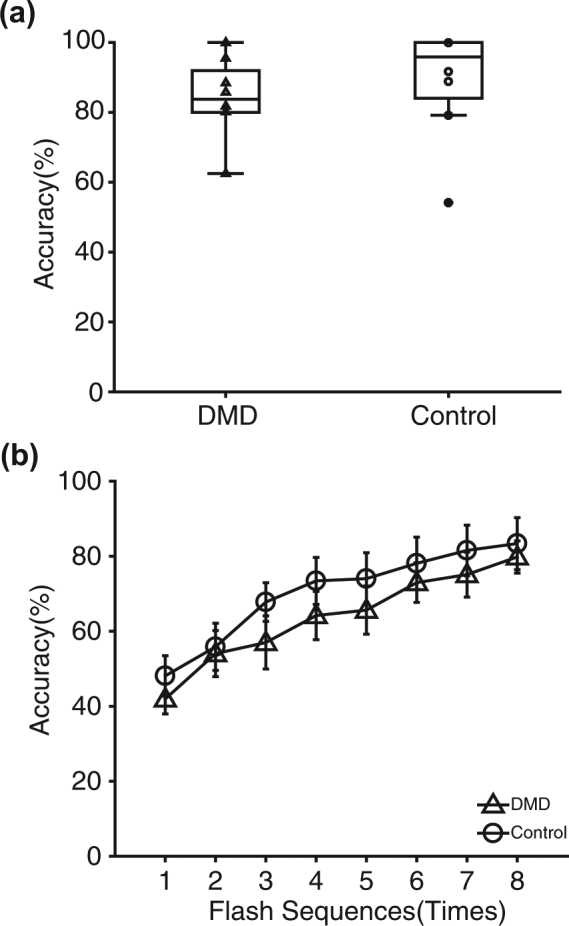
Online and offline accuracy in the BCI operation. **(a)** Online accuracy in the region-based two-step P300-BCI operation, which indicated the accuracy of both steps in total, was 79.8% for patients with DMD and 83.4% for controls; no significant difference was observed between them (*t*-test, p = 0.69). Each triangle (DMD) and circle (controls) indicates each data point. **(b)** Offline accuracy for both groups in each flashing sequence was plotted (triangle: DMD, and circle: controls).

The mean online accuracy in region-based two-step P300-BCI operation, which indicated the accuracy of both steps in total, was 79.8% for patients with DMD and 83.4% for controls, which were not significantly different (two tailed *t*-test, p = 0.69, df = 7; Fig. 2(a)).

Figure 2(b) shows the results of the offline analyses for both groups in each flashing sequence. We used two-way repeated-measures ANOVA with group (DMD vs. control) and accuracy for each sequence (1–8). The main effect of group was not significant (p = 0.0502, df = 1; Fig. 2(b)), but the main effect of number of sequence was significant (p = 3.71 × 10^−7^, df = 7; Fig. 2(b)). The interaction effect was not significant (p = 0.99, df = 7).

### Performance on character input

**Figure 3:**
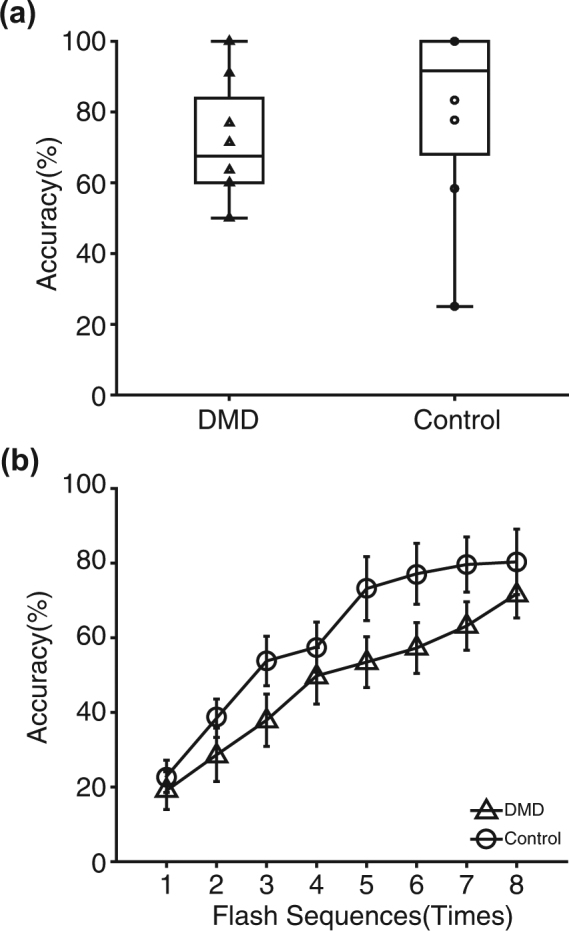
Performance on the character input. **(a)** Online accuracy of inputted characters (accuracy for the two-step procedure) in the DMD patients was 71.6% and that in the control was 80.6%, with no significant difference between them (*t*-test, p = 0.32). Each triangle (DMD) and circle (controls) indicates a data point. **(b)** Offline accuracy for both groups in each flashing sequence was plotted (triangle: DMD, and circle: controls).

The mean online accuracy of inputted characters (accuracy for the two-step procedure) in the DMD patients was 71.6% (9.34 bit/min) and that in the controls was 80.6% (11.24 bit/min) (Fig. 3(a)), which were not significantly different (two tailed *t*-test, p = 0.32, df = 7).

Figure 3(b) shows the results of the offline analyses for both groups under the two-step procedure. We tested a two-way repeated measures ANOVA with group (DMD vs. control) and the accuracy of each sequence (1–8). The main effects of group and the number of sequences were significant (group, p = 0.00081, df = 1; number of sequences, p = 9.9 × 10^−13^, df = 7; Fig. 3(b)). The interaction effect was not significant (p = 0.94, df = 7).

Note that the two-step procedure was accomplished only when the responses were correct for the first and second steps. The accuracy of the first step was 85.1% for patients with DMD and 83.9% for controls, and that for the second step was 74.6% for patients with DMD and 82.8% for controls.

### EEG waveforms

**Figure 4:**
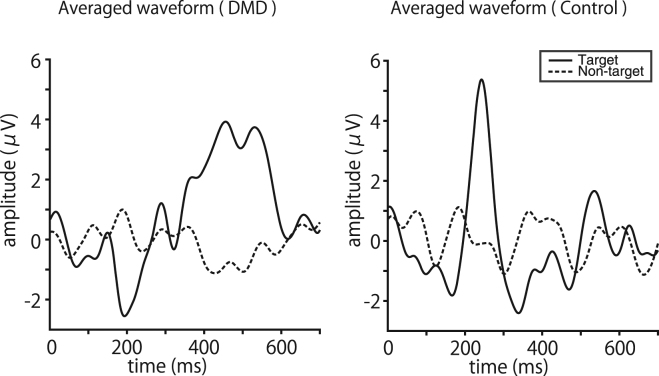
EEG waveforms in DMD and controls. EEG waveforms obtained from P4 electrodes, averaged for patients with DMD and controls, are shown. The 700 ms waveforms extracted from the onset of intensification were averaged. Solid lines indicate the target ERPs and dashed lines indicate non-target ERPs. In the controls, two positive peaks are shown at approximately 243 and 535 ms after the onset of stimuli, whereas in the DMD group, no clear peak corresponding to the early component was evident, but a blurred peak was seen at approximately 450–550 ms.

EEG waveforms obtained from P4 electrodes were averaged for patients with DMD and controls, as shown in Fig. 4, in which dashed lines indicate non-target ERPs, and solid lines indicate target ERPs. The P4 electrode was chosen specifically because, in previous studies, we showed the importance of electrode position in the operation of visual P300 BCIs with green/blue flicker stimuli^25,26^. The averaged waveforms of the target ERPs differed between the groups. In controls, two positive peaks were seen at approximately 243 and 535 ms after the onset of stimuli, which corresponded to the early and late components of P300, respectively, whereas in the DMD group, no clear peak corresponding to the early component was seen, but a blurred peak that may correspond to the late component was seen at approximately 450–550 ms.

## Discussion

We used a region-based two-step P300-BCI speller to evaluate accuracy in BCI operation and character input in bedridden DMD patients and able-bodied age- and gender-matched control participants. In online analyses, the DMD group achieved reliable accuracies, comparable to those of the control group in both BCI operation (Fig. 2(a)) and character input (Fig. 3(a)).

DMD is a neuromuscular disease that shows skeletal muscle atrophy throughout the body, involving the proximal muscle and causing gait disturbances^22^. DMD patients eventually progress to a bedridden state requiring full assistance. Patients with DMD show muscle weakness and muscle fatigue. After performing a tracheostomy, it becomes difficult for such patients to communicate verbally. In a clinical context, these DMD patients can be considered as candidate BCI users. In this study, we showed systematically, for the first time, that bedridden DMD patients were able to use the BCI speller.

It is important for bedridden DMD patients to be able to use the BCI system because deterioration of attention has been reported in these patients. For example, Piccini *et al*. used visuospatial attention tasks to compare DMD and control groups, showing that accuracy in the DMD group was significantly lower than in the controls, and that this was true not only in voluntary attention but also in automatic attention^27^.

Use of the region-based two-step system may have contributed to the successful operation of the BCI speller. In a previous study, we performed character-input experiments using a conventional row/column P300 speller for amyotrophic lateral sclerosis (ALS) patients under artificial ventilation control, but they could not control it well. However, when they used a region-based two-step BCI speller, the accuracy improved^20^. Similar to DMD patients, it has been reported that the ALS in later stages is often accompanied by deterioration in attention^28,29^. Thus, the region-based two-step system, which has a larger flashing area than a conventional visual array, may help in achieving better accuracies in these patients at an advanced disease stage.

Although the online analyses showed that the DMD group was able to achieve reliable accuracies comparable to those of the control group in both BCI operation and character input, further evaluation in the offline analyses showed some differences between the DMD and control groups. In the offline analyses of BCI input, two-way repeated measures ANOVA was used with group (DMD vs. control) and the accuracy for each sequence (1–8). The main effect of group was not significant, but the main effect of number of sequences was (Fig. 2(b)). In the offline analyses of character input, we also used ANOVA; the main effects of group and number of sequences were significant (Fig. 3(b)). Thus, for DMD patients, character input was slightly more difficult than BCI input than it was in the control group.

Further evaluation of the EEG waveforms also showed some differences between the DMD patients and controls. We averaged EEG waveforms obtained from P4 electrodes in both groups (Fig. 4). In the controls, two positive peaks were seen at approximately 243 and 535 ms after the onset of stimuli, corresponding to the early and late components of P300, respectively. In the DMD group, no clear peak corresponding to the early component was evident, but a blurred peak that may correspond to the late component was seen at approximately 450–550 ms.

P300 consists of the early component, occurring 150–300 ms after the presentation of a target stimulus, and the late component, occurring 300–500 ms after the presentation of the stimulus. It has been suggested that the early component corresponds to a stimulus-driven frontal attention mechanism and the late component is due to parietal activity^11^. Discrimination between a target stimulus and non-target stimuli at the time of task execution leads to the initiation of activity in the frontal lobe region, responding sensitively to attention efforts. The early component is generated when such stimulation is processed due to sufficient attention concentration, whereas the late component is generated when the activation of the resource for subsequent attention promotes a memory operation in the parietal region^30–32^. Reports have described that patients with frontal lobe lesions show a decrease in the amplitude of the early component and that frontal lobe integrity is necessary to generate the early component^33,34^. Thus, the lack of a clear peak corresponding to the early component in DMD patients may be related to their altered frontal lobe function.

The altered frontal lobe function in DMD patients has recently been discussed in light of their genetic background. DMD is accompanied by the loss of dystrophin, and dystrophin is known to play an important role in the development of the central nervous system in the foetal brain during pregnancy. In particular, it has a growth-promoting effect on neurons; expression of dystrophin in the brain contributes to synapse maturation and stabilisation^27,35,36^. Dystrophin is known to be highly expressed in the frontal lobe, together with the hippocampus and the cerebellum^27,36^. Thus, attenuation of the early component in DMD patients may be due to altered frontal lobe function, and the preserved late component is consistent with preserved parietal lobe function in DMD patients.

In this BCI study, using a region-based two-step P300-BCI speller, although the online analyses and EEG waveform analyses showed some differences between DMD patients and controls, likely due to altered frontal lobe function in DMD patients, in the online analysis, the DMD group achieved reliable accuracies, comparable to those of the control group in both BCI operation and character input. Thus, the P300-based BCI was operated successfully by individuals with DMD in an advanced stage, and these findings suggest that a BCI may be beneficial for patients with DMD.

## Methods

### Participants

Eight patients with DMD, aged 23–38 years (mean: 30.3), diagnosed by genetic testing and proof of dystrophin deficiency, were recruited as participants (Table 1). These patients had experienced walking disabilities before reaching 5 years old, and their current level of activities of daily living (ADL) was at a total assistance level; their modified Rankin Scale score was 5. Of these eight patients, five had undergone tracheostomies, and were under mechanical ventilation control, with tracheotomy positive pressure ventilation (TPPV). They communicated via lip reading, facial expressions, and with visual scanning boards. One of the patients used an alternative augmentative communication (AAC) device, called the Den-no-shin (Hitachi KE Systems, Chiba, Japan). Three patients had not undergone tracheostomies and were barely able to speak, due to muscle weakness and tongue hypertrophy. No obvious neurocognitive deterioration was observed. As a control group, eight able-bodied age- and gender-matched participants were recruited. Neither the patient group nor the control group had any BMI training beforehand.

The present study was approved by the institutional ethics committee at the National Rehabilitation Center for Persons with Disabilities. All participants provided written informed consent according to the institutional guidelines. All experiments were carried out in accordance with the approved guidelines.

### Experimental procedure

The P300 speller is a conventional method in BCI, which applies the oddball task paradigm that elicits P300-like responses^12^. Icons arranged in a row/column matrix are intensified randomly, and the subject is required to focus on one of the icons to be input, and to count the number of intensifications of the target icon. Focusing on the intensification of the target as a rare stimulus enhances the P300-like responses. In the original P300 speller^12^, 36 icons consisting of the English alphabet and 10 numerals or symbols were used and arranged in a 6 × 6 matrix.

In this experiment, we used a region-based, two-step P300 speller that used green/blue intensification (Fig. 1)^20^. This system uses a two-step paradigm, modified from previous research^37,38^. We divided the 6 × 9 matrix into six circled regions (i.e. 2 × 3 regions) including nine characters each. First, each region was intensified individually. Each circle flashed eight times, and the interval between the two flashes was 175 ms, consisting of 100 ms of intensification (green) and 75 ms of rest (blue). The traditional visual stimulus in the visual P300 BCI was a luminance change in the icons^12^, but we applied the green/blue colour combination, because we showed previously that adding a green/blue chromatic change to the luminance improved performance on the P300 speller^39^. The subject was verbally instructed to attend to the next target character to be input, and also to count the number of intensifications of the green/blue circle containing the target character. When a region that included the target character was selected, the speller matrix moved to the second step. The second step used 3 × 3 regions with one character each, and each region was again intensified. The subject was again verbally instructed to attend to the next target character to be input, and also to count the number of intensifications of the green/blue circle with the target character.

Four of the eight DMD patients rested in a supine position on a bed, and a liquid crystal display (LCD), on which visual stimuli were presented, was set ~100 cm from their eyes. The other four DMD patients and the able-bodied control participants sat in a chair, and the LCD was also set ~100 cm from their eyes. The size of the LCD was 21.7 cm in height and 38.5 cm in width.

### EEG recordings and BCI analyses

EEG data were recorded from eight channels (Fz, Cz, Pz, P3, P4, Oz, PO7, PO8) using an in-house cap with solid-gel electrodes^40^. All channels were grounded to AFz and referenced to Fpz. We used an in-house amplifier, with which the sampling rate was 1024 Hz, the precision of the analogue-to-digital converter was 24 bit, and the hardware notch filter was 50 Hz. The recorded signals were band-pass filtered (0.1–50 Hz) and the data were down-sampled to 21 Hz for analyses.

To identify the region at which the subject was gazing, first, we applied a preparation session that derived a feature vector to discriminate target and non-target for each individual. We used a specifically prepared panel that included 3 × 3 regions, which include nine characters each, during the preparation session. We asked the subjects to attend to nine regions each to derive the feature vector. Each region was intensified eight times, thus 72 segmented EEG data for target and 576 segmented EEG data for non-target were used. A total of 800 ms EEG data were segmented according to the timing of the intensification onset to derive the feature vector. The first 100 ms, just prior to flash onset, were used to correct the baseline, and the remaining 700 ms were used for classification. Because the 700 ms baseline-corrected EEG corresponded to 15 data points, and the data were collected from eight EEG channels, the feature vector had 120 dimensions. Fisher’s linear discriminant analysis was used to compute the feature vector. Target and non-target characters were classified using the feature vector during the test session. The maximum of the summed scores, which was the result of this classification, was used to determine the region to which the subjects were attending. The classification processes were applied to both online and offline analyses.

### EEG waveform analyses

We evaluated the elicited EEG waveforms. EEG signals obtained from P4 electrodes were averaged for patients with DMD and controls. We specifically focused on the P4 electrode because, in previous studies, we showed the importance of this electrode position in the operation of visual P300 BCIs with green/blue flicker stimuli^25,26^. The 800 ms waveforms extracted from the onset of intensification were averaged. The first 100 ms just prior to the onset of intensification was used for baseline correction.
